# *KMT2C* mutation in a Chinese man with primary multidrug-resistant metastatic adenocarcinoma of rete testis: a case report

**DOI:** 10.1186/s12894-022-01075-8

**Published:** 2022-08-09

**Authors:** Yue Zhang, Xiaoyan He, Hui Gao

**Affiliations:** 1 Department of Oncology, General Hospital of Western Theatre Command, No. 270, Tianhui Road, Rongdu Avenue, Jinniu District, Chengdu, 610000 Sichuan People’s Republic of China; 2 Department of Pathology, General Hospital of Western Theatre Command, Chengdu, Sichuan People’s Republic of China

**Keywords:** Adenocarcinoma of rete testis, KMT2C, Multi-drug resistance, Mutation

## Abstract

**Background:**

Adenocarcinoma of the rete testis (AORT) is an extremely rare malignant tumor with poor prognosis and limited responsiveness to traditional chemotherapy. Few previous studies have focused on the molecular mechanisms underlying therapy resistance in AORT and further scrutiny is required to enable searches for targeted drugs to guide treatment selection.

**Case presentation:**

The current case concerns a 55-year-old man with AORT who presented with isolated bone metastasis at initial diagnosis and experienced rapid disease progression after multi-line platinum-based combination chemotherapy. Next-generation sequencing revealed a novel somatic lysine methyltransferase 2C (*KMT2C*) c.5605 T > C mutation in exon 36 with an abundance of 49.27%. The patient received antiangiogenic drug treatment for 2 months but this was discontinued due to unacceptable anorexia and nausea. He survived for 12 months after diagnosis.

**Conclusion:**

A potential correlation between AORT primary multi-drug resistance and *KMT2C* mutations is implied. Further studies are needed to determine the efficacy of PARP1/2 inhibitors for tumors with *KMT2C* mutations.

## Background

Adenocarcinoma of the rete testis (AORT) is a very rare malignant tumor originating from the testis hilum. The disease is most prevalent in males aged 17–91 with a median age of 57 years [1]. Recent reports of AORT have increased but most cases have focused on the choice of postoperative chemotherapy since poor responses to system therapy are elicited. Little is known concerning genomic drivers or molecular targets in AORT.

A summary of more than 33,000 cases conducted by the National Cancer Institute Genomic Data Commons (GDC) has revealed that lysine methyltransferase 2C (*KMT2C*) is the seventh most frequently mutated gene in cancer [2]. It is a member of the myeloid/lymphoid or mixed-lineage leukemia (MLL) family and encodes histone methyltransferase 2C. KMT2C protein binds and methylates the enhancer region of histone 3 lysine 4 (H3K4) and promotes the recruitment of transcriptional activators to participate in DNA replication restart [3, 4]. *KMT2C* plays a tumor-suppressing role and deletions or mutations which inactivate the gene are frequently found in solid tumors. Deletion of the catalytic core of the SET (Su(var)3‐9, Enhancer of Zeste, Trithorax) domain in *KMT2C* induced hyperproliferation of cells and formation of urothelial tumors [5], but knockout of the entire gene was lethal in mice [6].

The current report concerns a Chinese man with rapidly progressive metastatic AORT, who exhibited primary resistance to platinum-based two-drug combination chemotherapy. This patient had a somatic *KMT2C* mutation that has not been previously described. However, insufficient clinical evidence currently exists to guide the recommendation of drugs targeting this gene mutation.

## Case presentation

**Fig. 1:**
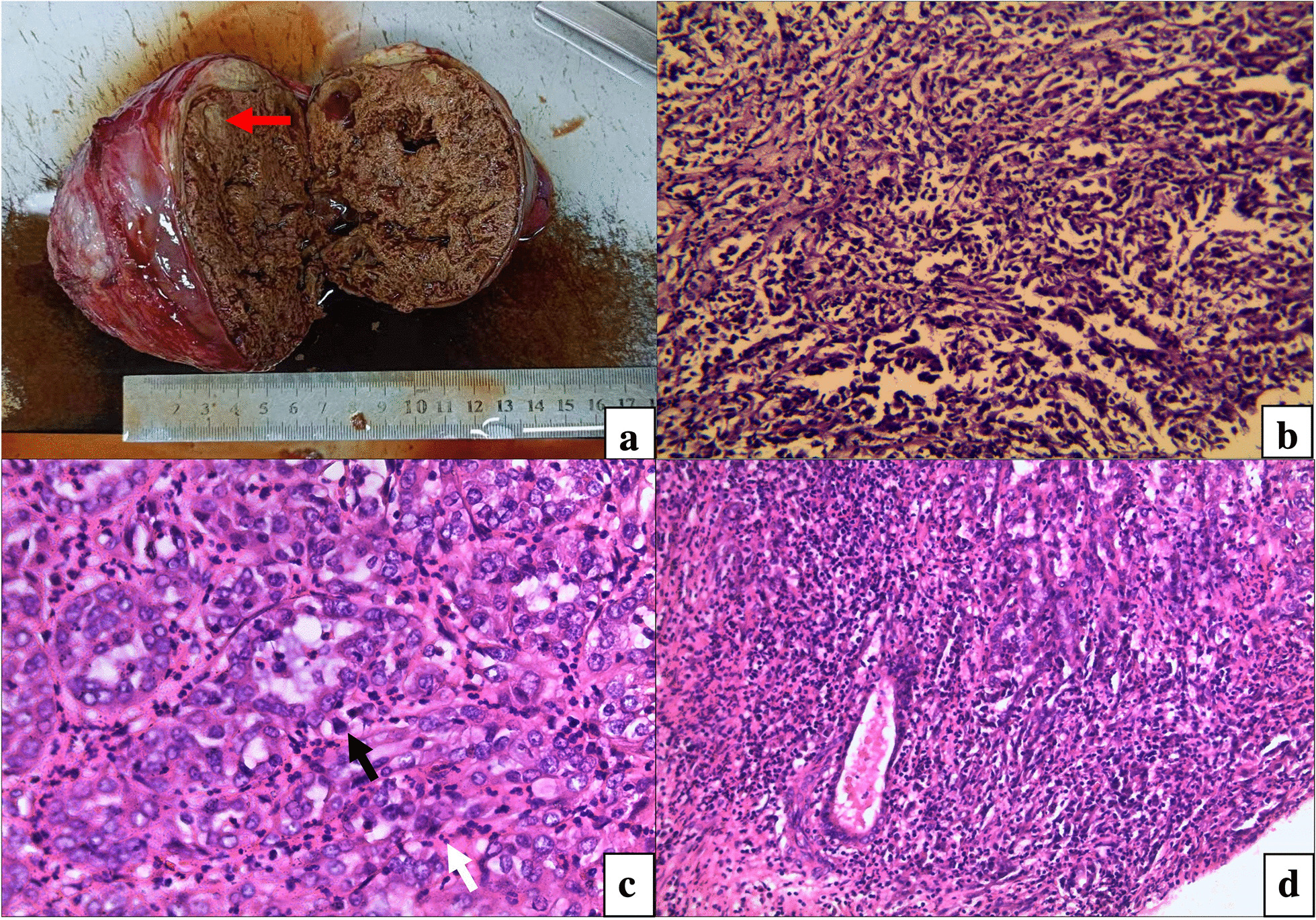
Gross and H&E staining specimens: **a** showed a tumor located in the hilum of the testis with a maximum diameter of 13.0 cm. It was close to the testicular tunica albuginea with an unclear boundary, as indicated by the red arrow. **b** Showed that tumor cells in this area were spiked and constituted slit-like branched tubules, with hyperchromatic and pleomorphic nuclei (magnified ×200). **c** Showed tumor cells arranged in an acinar-like structure. Normal and abnormal mitoses were indicated by white and black arrows, respectively (magnified ×400). **d** Showed atrophied seminiferous tubules surrounded by tumor cells (magnified ×200)

**Fig. 2:**
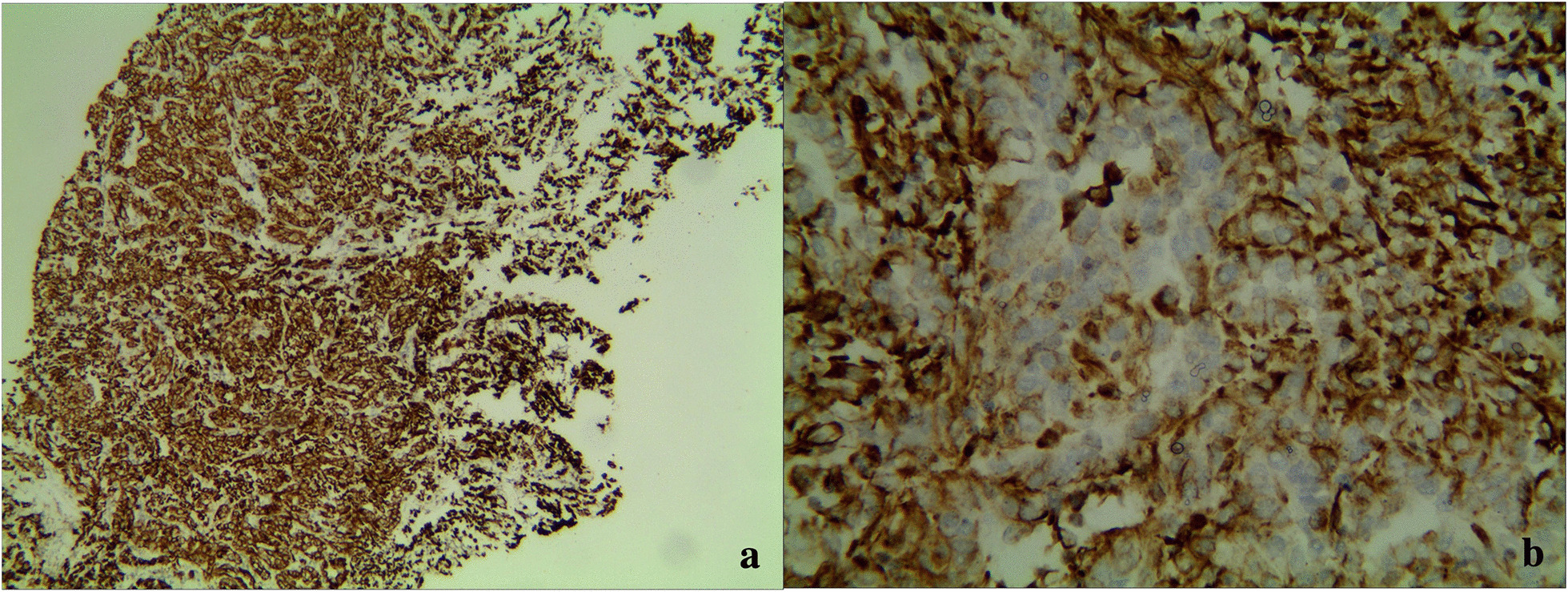
Immunohistochemistry showed high expression of CK7 in the cytoplasm and membrane of tumor cells (**a**: magnified ×100; **b**: magnified ×400)

The 55-year-old male patient was diagnosed on March 26th, 2021, following a chief complaint of "left testis swelling for 16 years and right hip pain for 1.5 months". Physical examination revealed a hard mass in the left testis, approximately 10 cm in diameter, without tenderness and invading the skin. The patient had no history of other diseases but had smoked about 20 cigarettes a day for more than 30 years. Neither his parents nor his children had a history of cancer. Computer tomography (CT) showed a huge low-density mass, 10.1 × 8.6 cm, located in the left testis and accompanied by marginal calcification, and a large soft tissue mass, 15.0 × 12.0 cm in the right iliac fossa, accompanied by bone destruction. There were no metastases in the groin, abdominal cavity, retroperitoneum, or lungs. Laboratory tests did not reveal elevated tumor markers, including α-fetoprotein (AFP), β-human chorionic gonadotropin (β-hCG), lactate dehydrogenase (LDH), carbohydrate antigen 199 (CA199), CA125, carcinoembryonic antigen (CEA) or prostate-specific antigen (PSA). Mass biopsy of the right iliac fossa demonstrated invasion of moderately and poorly differentiated adenocarcinoma. A radical resection of the left testis was performed on April 12 and gross specimens showed a tumor located in the hilum of the testis with a maximum diameter of 13.0 cm, accompanied by necrosis. The tumor was close to the testicular tunica albuginea with an unclear boundary. The texture was soft, gray-white and gray-yellow in color and remaining testicular tissue was sponge-like (Fig. 1a). Microscopically, the tumor cells were spiked with hyperchromatic and pleomorphic nuclei. They had formed largely slit-like branched tubules with a few papillary and solid areas which had grown into one another in varying proportions (Fig. 1b). We could also find acinar-like structures, many mitosis (Fig. 1c), interstitial fibrosis accompanied with chronic inflammatory cell infiltration and atrophic spermatogenic tubules surrounded by tumor cells (Fig. 1d). There were vascular invasions but no nerve bundle invasions. The immunohistochemistry results showed positive cytokeratin 7 (CK7; Fig. 2) and Ki-67 (approximately 40%) but negative vimentin, synaptophysin (Syn), chromogranin A (cgA), cluster of differentiation 56 (CD56), Wilms tumor 1 (WT-1), calretinin (CR), thyroid transcription factor 1 (TTF-1), caudal type homeobox transcription factor 2 (CDX-2), CD30, CK20, CK5/6, CEA, AFP, P63, CD117, β-hCG, and Villin.

**Fig. 3:**
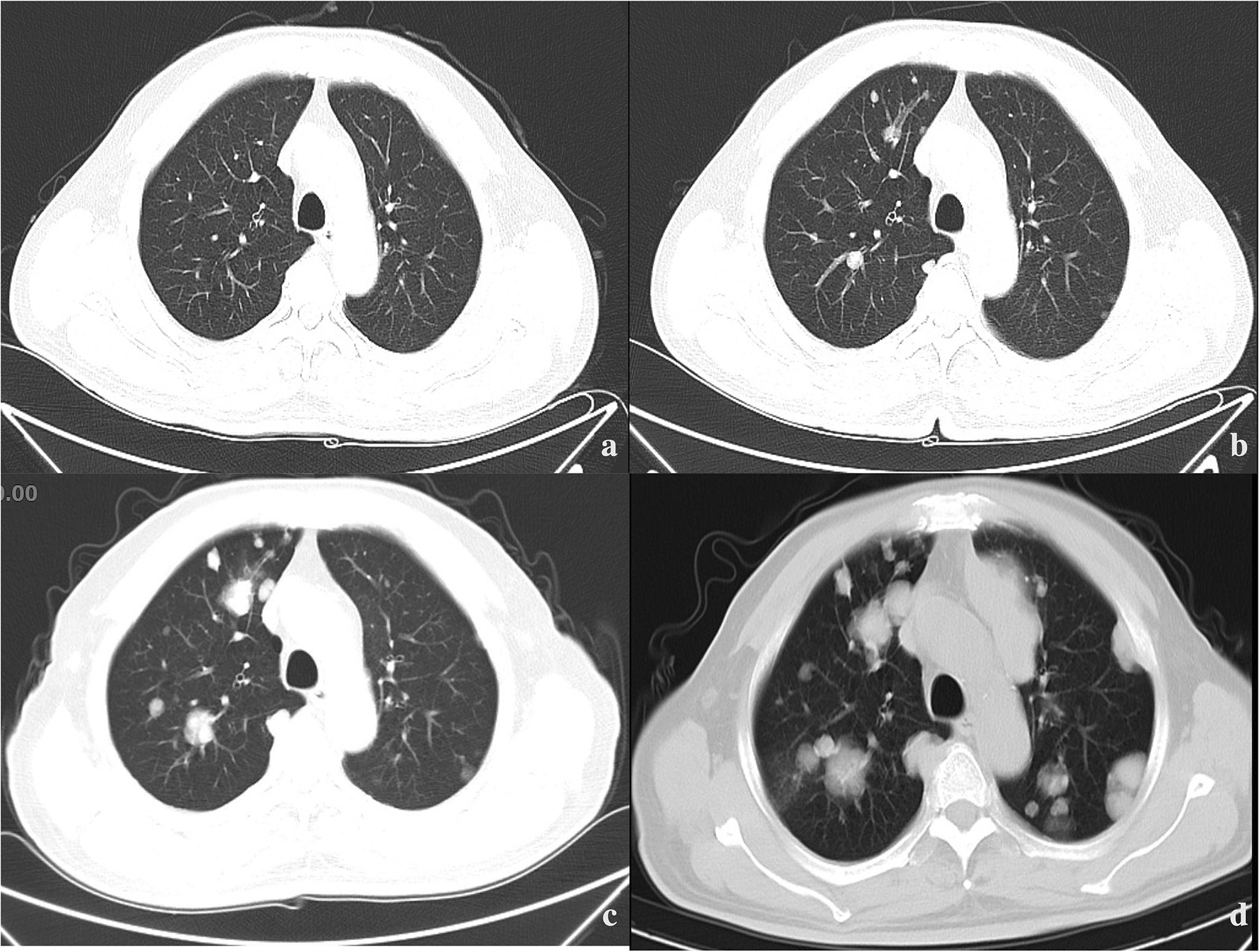
CT images of pulmonary: **a** showed no lung metastases when the patient was diagnosed on March 2021; **b** showed multiple metastases located in lungs on June 2021, after orchiectomy and 2 cycles of chemotherapy; **c** showed progressed lung metastases on August 2021, after 2 cycles of second- or third-line chemotherapy; **d** showed recently progressed lung metastases on January 2022

**Fig. 4:**
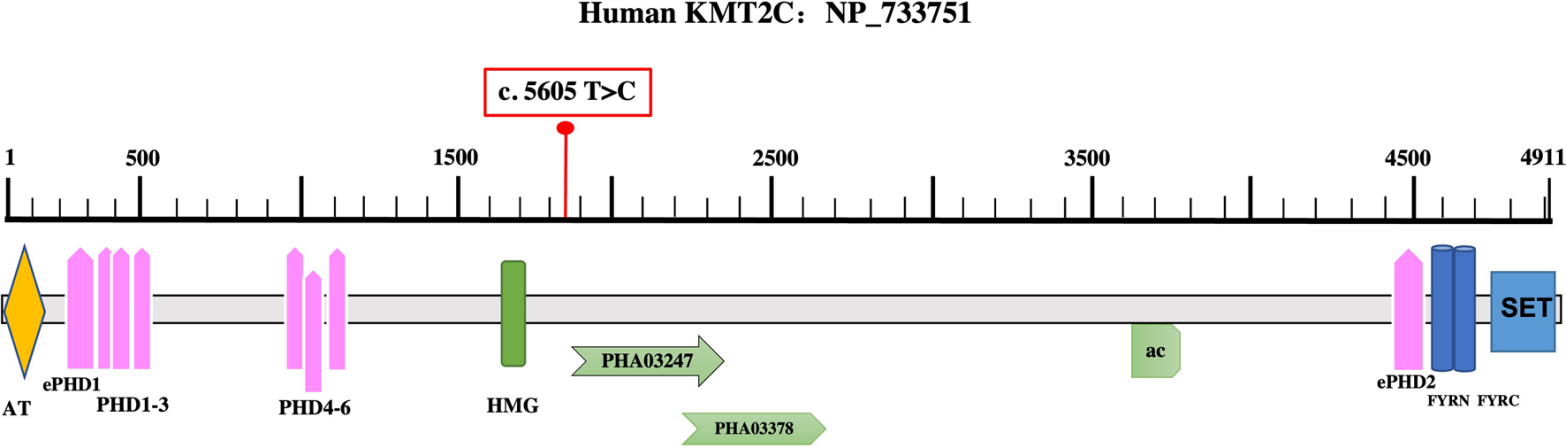
Schematic diagram of KMT2C domains location of the c.5605 T > C mutation

Post-orchiectomy, the patient received 2 cycles of nab-paclitaxel combined with nedaplatin on May 6 and May 28 2021, and intensity-modulated radiation therapy (IMRT) to the metastasis with a dose of 2,640 cGy in 220 cGy per fraction. However, he developed multiple metastases in both lungs and in the 12th thoracic vertebra two months after the beginning of first-line chemotherapy (Fig. 3). Treatment was changed to one cycle of irinotecan combined with capecitabine. On July 14th 2021, a repeat CT showed progressive lung metastases. Next-generation sequencing (NGS) was performed targeting 700 genes associated with solid tumors to identify therapeutic targets. A somatic lysine methyltransferase 2C (*KMT2C*) c.5605 T > C mutation was found in exon 36 with an abundance of 49.27% (Fig. 4). No targeted drugs were available and one cycle of etoposide combined with cisplatin and cyclophosphamide was administered. Unfortunately, the patient suffered grade 4 neutropenia, and chest and abdominal CT revealed continually progressing lung metastases on August 18th, 2021 (Fig. 3). Subsequently, the patient was switched to 2 cycles of Anlotinib which kept the disease stable until October 29th 2021 when the patient refused to continue the drug due to unacceptable anorexia and nausea. He died on April 6th, 2022, having survived for 12 months since diagnosis.

## Discussion and conclusions

To the best of our knowledge, this is the first report of a somatic *KMT2C* c.5605 T > C mutation associated with AORT. AORT is a very rare tumor with a poor prognosis and fewer than 100 cases have been reported in the literature [7]. The most common symptom is painless scrotal mass and 19% of patients present with symptoms related to metastasis, such as hip or back pain. As a result of the atypical symptoms and aggressive features of AORT, many patients have developed distant metastases prior to diagnosis and 3- and 5-year disease-free survival rates are only 49% and 13%, respectively. Patients with a tumor diameter < 5 cm have better survival rates than patients with larger tumors [8, 9].

As regards the current case, the chief complaint of "left testis swelling for 16 years” is unlikely to be compatible with this aggressive AORT tumor. Pathomorphological examination and negative expression of β-hCG, CD30, CD117 and AFP completely excluded germ cell tumors, such as teratoma, and we guess that this patient has had a benign disease for many years, such as hydrocele of tunica vaginalis, that also exhibits testis swelling, but has not sought medical help for reasons such as privacy or embarrassment. Regrettably, the opportunity to confirm the cause of this chief complaint no longer exists following the patient’s death.

The pathological and immunohistochemical features of AORT have not been established due to the extremely rare incidence of the disease. Most cases show a mixed morphology of papillary, glandular and solid tumors. AE1/AE3 is expressed by 96% of AORT patients, epithelial membrane antigen (EMA) by 84%, CK 7 by 83% and vimentin by 81%. Malignant mesothelioma (MM) shares overlapping morphology and immunohistochemical markers (such as WT-1, CR, CK 5/6 and paired box 8 [PAX8]) with AORT and represents the most significant differential diagnosis [7]. However, unlike MM, AORT is mainly located on the testicular hilum and is continuous with the normal rete testis epithelium (Fig. 1d). In addition, AORT has more slit-like branched tubules and greater nuclear pleomorphism than MM, in which these features are rare. Electron microscopy can be used as a final technology to distinguish the two diseases since AORT has fewer short, blunt microvilli than MM [10, 11].

The diagnosis of AORT is generally based on the modified criteria first described by Nochomovitz and Orenstein [12] in 1984: (1) the tumor is located in the testicular hilum; (2) the microscopic morphology differs from that of other testicular or paratesticular tumors; (3) there is no histologically similar primary tumor outside the scrotum and (4) other possible tumors, such as MM, should be excluded by immunohistochemical analyses. Other tumors, such as MM, germ cell tumors and sex cord-stromal tumors, were ruled out in the present case as far as possible, based on pathomorphological and immunohistochemical analyses. The tumor was finally diagnosed as AORT.

Orchiectomy currently represents the primary treatment modality for AORT, but retroperitoneal lymph node dissection remains under consideration. In a retrospective study of 78 patients conducted by Khaleel I. Al-Obaidy [7], patients who underwent retroperitoneal lymph-node dissection survived slightly longer than those who did not, but a statistically significant difference was not achieved (*p* = 0.2). In addition, no statistically significant differences in survival between patients who received adjuvant chemotherapy and those who did not were found (*p* = 0.6), although Shunsuke Owa et al. [13] reported that a patient with metastatic AORT did not progress for 7 months and survived for 20 months after 7 cycles of platinum-based combined chemotherapy. Most previous reports indicate poor responses to traditional chemotherapy by metastatic cases [14]. Consistent with these findings, the current patient developed multiple metastases to the lung and thoracic vertebra during the two cycles of platinum-based combined chemotherapy and showed resistance to second- and third-line chemotherapy regimens.

The mechanisms whereby AORT achieves resistance to chemotherapy have not previously been described. NGS of the current patient’s tumor tissue demonstrated a somatic c.5605 T > C mutation in *KMT2C* gene exon 36, with an abundance of 49.27%. The association of *KMT2C* with cancer was first discovered through tumor gene-sequencing studies which revealed *KMT2C* deletions as the most common chromosomal abnormality in acute myeloid leukemia. Thus, a tumor suppressor role was suspected for *KMT2C* [15, 16]. The TCGA-BRCA study has also shown *KMT2C* to be one of the most frequently mutated genes in ER-positive breast cancer and patients with low *KMT2C* expression had longer overall survival (OS) [17].

In addition, mutations in *KMT2C* are also involved in the genetic mechanism of primary resistance to chemotherapy and may be a potential chemosensitivity biomarker in childhood acute myeloid leukemia [18]. Breast cancer patients with somatic *KMT2C* mutations had worse OS and disease-free survival (DFS) than those with wild-type *KMT2C*. However, patients with low *KMT2C* expression had much longer DFS than those with high expression if they received doxorubicin, cisplatin, and carboplatin chemotherapy [19]. Soo-Jeong Cho et al. demonstrated that *KMT2C* knockout promoted the epithelial-to-mesenchymal transition (EMT) and induced chemoresistance in diffuse-type gastric adenocarcinomas and *KMT2C* re-expression reversed chemotherapy resistance via promotion of DNA damage and apoptosis [20]. However, the c.5605 T > C mutation is located in a sequence encoding an intrinsically disordered peptide region and its effect on KMT2C function is unclear. We speculate that the c.5605 T > C mutation in *KMT2C* gene exon 36 may result in inactivation of the protein product and contribute to primary chemoresistance in the current patient.

Recent studies have suggested a role for *KMT2C* in DNA damage repair (DDR). Functional deficiency of the KMT2C protein predicted a good response to Poly-(ADP)-ribose polymerase 1/2 (PARP1/2) inhibitor therapy. Rampias et al. [21] indicated that bladder cancer cells with low levels of KMT2C expression have heightened sensitivity to PARP1/2 inhibitor therapy. Chang et al*.* further proved that KMT2C promoted DDR through direct recruitment to DNA damage sites and that *KMT2C* mutations sensitized non-small- cell lung cancer (NSCLC) to PARP1/2 inhibitors by disrupting homologous recombination (HR)-mediated DNA repair [22]. Hence, we look forward to more evidence supporting the administration of PARP1/2 inhibitors in cases of tumors with *KMT2C* mutations.

In summary, the current case demonstrates a potential correlation between primary multi-drug resistance AORT and *KMT2C* mutation. Further studies are needed to prove this association and explore the efficacy of PARP1/2 inhibitors for tumors with *KMT2C* mutations.

## Data Availability

Not applicable.

## Abbreviations

AFP: α-Fetoprotein
AORT: Adenocarcinoma of the rete testis
β-hCG: β-Human chorionic gonadotropin
CA: Carbohydrate antigen
CD: Cluster of differentiation
CDX-2: Caudal type homeobox transcription factor 2
CEA: Carcinoembryonic antigen
cgA: Chromogranin A
CK: Cytokeratin
CR: Calretinin
CT: Computer tomography
DDR: DNA damage repair
DFS: Disease-free survival
EMA: Epithelial membrane antigen
H3K4: Histone 3 lysine 4
IMRT: Intensity-modulated radiation therapy
KMT2C: Lysine methyltransferase 2C
LDH: Lactate dehydrogenase
MLL: Mixed-lineage leukemia
MM: Malignant mesothelioma
NGS: Next-generation sequencing
OS: Overall survival
PARP1/2: Poly-(ADP)-ribose polymerase1/2
PSA: Prostate-specific antigen
SET: Su(var)3‐9, Enhancer of Zeste, Trithorax
Syn: Synaptophysin
TTF-1: Thyroid transcription factor 1
WT-1: Wilms tumor 1
